# First-Principles Calculations on Structural Property and Anisotropic Elasticity of *γ*_1_-Ti_4_Nb_3_Al_9_ under Pressure

**DOI:** 10.3390/ma11102025

**Published:** 2018-10-18

**Authors:** Xianshi Zeng, Rufang Peng, Yanlin Yu, Zuofu Hu, Yufeng Wen, Lin Song

**Affiliations:** 1 Research Center of Laser Fusion, China Academy of Engineering Physics, Mianyang 621900, China; zengxueliang@163.com; 2 State Key Laboratory for Environment-friendly Energy Materials, Southwest University of Science and Technology, Mianyang 621010, China; rfpeng2006@163.com; 3 School of Mathematical Sciences and Physics, Jinggangshan University, Ji’an 343009, China; yuyanlin 121@163.com (Y.Y.); huzuofu@outlook.com (Z.H.); 4 State Key Laboratory of Solidification Processing, Northwestern Polytechnical University, Xi’an 710072, China; songlin@nwpu.edu.cn

**Keywords:** *γ*1-Ti_4_Nb_3_Al_9_, structural property, anisotropic elasticity, pressure effect, first-principles

## Abstract

The effect of pressure on the structural property and anisotropic elasticity of $\gamma_{1}$-Ti${}_{4}$Nb${}_{3}$Al${}_{9}$ phase has been investigated in this paper by using first-principles calculations. The obtained bulk properties at zero pressure are in good agreement with the previous data. The structural property and elastic constants under pressures up to 40 GPa have been obtained. According to the elastic stability conditions under isotropic pressure, the phase is found to be mechanically stable under pressures up to 37.3 GPa. From the obtained elastic constants, the elastic moduli, anisotropic factors and acoustic velocities under different pressures have also been obtained successfully together with minimum thermal conductivities and Debye temperature. It is shown that the ductility of the phase is improved and its anisotropy and Debye temperature are enhanced with increasing the pressure.

## 1. Introduction

High Nb containing γ-TiAl-based alloys possess comparable high temperature properties to the conventional Ni-based superalloys for turbine disk applications, whereas the corresponding mass density is only half that of conventional Ni-based superalloys [1]. These attractive properties make high Nb containing γ-TiAl-based alloys the most promising alternative with respect to the conventional Ni-based superalloys. Currently, the devolopment of high Nb containing γ-TiAl-based alloys has become one of the important directions of lightweight high temperature structural materials. Considerable research efforts have been devoted to high Nb containing γ-TiAl-based alloys.

Excellent properties of high Nb containing γ-TiAl-based alloys are closely related to their phase structures. It has been proven that Nb atoms in γ-TiAl substitutes for Ti atoms and preferentially occupies the Ti sublattice [2]. Some experimental studies on phase relationship for the alloys showed that the continuous ordering of Nb atoms in the Ti sublattice of γ-TiAl appears with increasing Nb content, resulting in the Nb atoms to occupy specific Ti sublattices to finally form a new ternary ordered phase named $\gamma_{1}$[3,4,5,6,7,8,9]. The $\gamma_{1}$ phase has been confirmed as a stable phase [4,5,6,7,8,9]. It has the determined chemical formula of Ti${}_{4}$Nb${}_{3}$Al${}_{9}$ and the tetragonal structure with space group P4/mmm, lattice constant *a* in the range of 5.58–5.84 Å and lattice constant *c* in the range of 8.15–8.45 Å [4,5]. It has been revealed that the precipitation of $\gamma_{1}$-Ti${}_{4}$Nb${}_{3}$Al${}_{9}$ phase can inhibit dislocation motion on the slipping plane {111} of the γ-TiAl matrix, and thus improve the strength of high Nb containing γ-TiAl-based alloys [7,8,9,10]. The alloys with $\gamma_{1}$ phase have higher room-temperature and high-temperature strengths than those without $\gamma_{1}$ phase [11]. This makes it possible to develop advanced $\gamma_{1}$ plus γ alloys for higher service temperatures.

Knowledge of elastic constants is crucial to soundly understand the mechanical properties of materials. They are fundamental and indispensable parameters to describe the mechanical properties. The evident and direct application of them is the evaluation of elastic strains or energies in the materials under external force, internal stress, thermal stress, etc. Values of elastic constants provide valuable information on the structural stability, bonding characteristic between adjacent atomic planes and anisotropic character of the bonding. The elastic moduli determined from the elastic constants can be employed to assess some mechanical properties of the materials such as ductility/brittleness, hardness, strength, and so on [12]. The plastic properties of the materials are also closely related to the shear moduli along the slip planes of mobile dislocations because these dislocations can dissociate into partials with a spacing determined by the balance between the planar fault energy and the repulsive elastic force. Moreover, elastic properties are closely associated with other properties of the materials such as acoustic velocity, thermal conductivity, Debye temperature, and so on.

Although the ground-state elastic properties of some constituent phases in high-Nb containing TiAl alloys have already been investigated in both theory and experiment [13,14,15,16,17,18,19,20,21,22], the elastic properties of various phases under pressure have rarely been studied. Up to now, the structural and elastic properties of γ-TiAl, $\alpha_{2}$-Ti${}_{3}$Al and B19-TiAl phases under pressure have been investigated by using first-principles calculations [23,24,25,26]. However, the structural and elastic properties of $\gamma_{1}$-Ti${}_{4}$Nb${}_{3}$Al${}_{9}$ phase under pressure have not been investigated theoretically yet to our knowledge. It is well known that pressure is an important variable to tune the properties of materials. This attracts us to study the pressure dependence of the structural and elastic properties of $\gamma_{1}$-Ti${}_{4}$Nb${}_{3}$Al${}_{9}$ phase. Therefore, the first-principles calculations shall be taken in this work to study the structural and elastic properties of $\gamma_{1}$-Ti${}_{4}$Nb${}_{3}$Al${}_{9}$ phase under pressure.

## 2. Materials and Methods

### 2.1. Crystal Structure of $\gamma_{1}$-Ti${}_{4}$Nb${}_{3}$Al${}_{9}$ Phase

**Figure 1:**
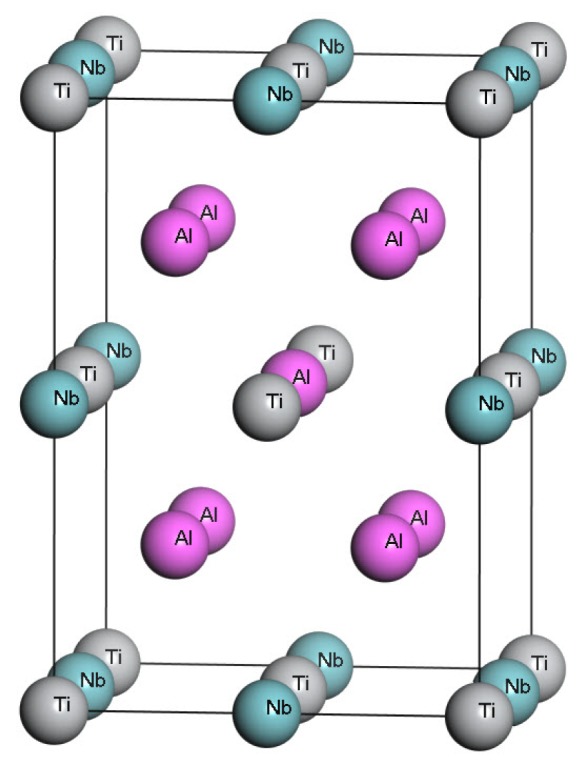
The unit cell of $\gamma_{1}$-Ti${}_{4}$Nb${}_{3}$Al${}_{9}$ phase.

The crystal structure of $\gamma_{1}$-Ti${}_{4}$Nb${}_{3}$Al${}_{9}$ phase is shown in Figure 1. The unit cell of the phase consists of eight body-centered tetragonal primitive cells of γ-TiAl. There are four Ti, three Nb and nine Al atoms in the unit cell. The corresponding atom occupation is (0, 0, 0), (0.5, 0, 0.5), (0, 0.5, 0.5) and (0.5, 0.5, 0) sublattice sites for four Ti atoms, (0.5, 0, 0), (0, 0.5, 0) and (0, 0, 0.5) sublattice sites for three Nb atoms, (0.5, 0.5, 0.5), (0.25, 0.25, 0.25), (0.25, 0.75, 0.25), (0.75, 0.25, 0.25), (0.75, 0.75, 0.25), (0.25, 0.25, 0.75), (0.25, 0.75, 0.75), (0.75, 0.25, 0.75) and (0.75, 0.75, 0.75) sublattice sites for nine Al atoms [3].

### 2.2. Computational Details

First-principles calculations were conducted within the framework of density functional theory (DFT) as implemented in the Vienna Ab initio Simulation Package (VASP) [27,28,29]. The projector augmented wave (PAW) method was used to describe the ion-electron interaction [30,31]. The Perdew, Burke and Ernzerhof (PBE) generalized gradient approximation (GGA) was used for the treatment of the exchange-correlation functional [32,33]. The valence electron configurations are 3s${}^{2}$3p${}^{6}$3d${}^{2}$4s${}^{2}$ for Ti, 4s${}^{2}$4p${}^{6}$4d${}^{4}$5s${}^{1}$ for Nb and 3s${}^{2}$3p${}^{1}$ for Al. The plane wave cut-off energy was specified to be 600 eV. The convergence criterion for electronic self-consistency loop was fixed to be $10^{-6}$ eV/atom. The Monkhorst–Pack scheme was used to construct the *k*-point meshes for the Brillouin zone sampling [34]. A 9×9×7*k*-points grid was used. To study the pressure effect on the structural property of $\gamma_{1}$-Ti${}_{4}$Nb${}_{3}$Al${}_{9}$ phase, the unit cell of the phase at different pressures up to 40 GPa was fully relaxed with respect to the volume, shape and internal atomic positions until the atomic forces of less than 0.01 eV/Å.

### 2.3. Calculations of Elastic Constants and Related Properties

The tetragonal $\gamma_{1}$-Ti${}_{4}$Nb${}_{3}$Al${}_{9}$ phase has six independent single crystal elastic constants $C_{11}$, $C_{12}$, $C_{13}$, $C_{33}$, $C_{44}$ and $C_{66}$. Starting from the optimized unit cell at a given pressure *P*, these six elastic constants were determined in this study by taking the strain–stress relationship method embedded in the VASP. In the method, the elastic constants are defined as the first derivatives of the stresses with respect to the strain tensor [35]. The elastic tensor is determined by performing six finite distortions of the lattice and deriving the elastic constants from the strain–stress relationship. The elastic tensor is calculated both for rigid ions, as well, as allowing for relaxation of the ions. The ionic contributions are determined by inverting the ionic Hessian matrix and multiplying with the internal strain tensor [36]. The final elastic constants include both the contributions for distortions with rigid ions and the contributions from the ionic relaxations. From the calculated elastic constants $C_{ij}$s, the six independent elastic compliances $S_{11}$, $S_{12}$, $S_{13}$, $S_{33}$, $S_{44}$ and $S_{66}$ of the $\gamma_{1}$-Ti${}_{4}$Nb${}_{3}$Al${}_{9}$ phase are determined according to the following relations:

(1) $$\begin{array}{c} S_{11}=\frac{1}{2}\left[\frac{C_{33}}{C}+\frac{1}{C_{11}-C_{12}}\right],\;\;S_{12}=\frac{1}{2}\left[\frac{C_{33}}{C}-\frac{1}{C_{11}-C_{12}}\right],\;\;S_{13}=-\frac{C_{13}}{C},\\ S_{33}=\frac{C_{11}+C_{12}}{C},\;\;S_{44}=\frac{1}{C_{44}},\;\;S_{66}=\frac{1}{C_{66}},\;\;C=\left(C_{11}+C_{12}\right)C_{33}-2C_{13}^{2}. \end{array}$$

In terms of the obtained $C_{ij}$s and $S_{ij}$s, the bulk (*B*), shear (*G*) and Young’s (*E*) moduli and Poisson’s ratio (ν) are obtained by using Voigt–Reuss–Hill approximation for $\gamma_{1}$-Ti${}_{4}$Nb${}_{3}$Al${}_{9}$ phase [37,38,39]:

(2) $$\begin{array}{c} B=\frac{B_{V}+B_{R}}{2},\;\;G=\frac{G_{V}+G_{R}}{2},\;\;E=\frac{9BG}{3B+G},\;\;\nu =\frac{3B-2G}{6B+2G},\\ B_{V}=\frac{2C_{11}+2C_{12}+4C_{13}+C_{33}}{9},\;\;B_{R}=\frac{1}{2\left(S_{11}+S_{12}\right)+4S_{13}+S_{33}},\\ G_{V}=\frac{2C_{11}-C_{12}-2C_{13}+C_{33}+6C_{44}+3C_{66}}{15},\;\;G_{R}=\frac{15}{8S_{11}-4S_{12}-8S_{13}+4S_{33}+6S_{44}+3S_{66}}. \end{array}$$

The percentage anisotropy in bulk ($A_{B}$) and shear ($A_{G}$) moduli and the universal anisotropy index ($A^{U}$) are further obtained by [40,41]

(3) $$\begin{array}{c} A_{B}=\frac{B_{V}-B_{R}}{B_{V}+B_{R}},\;\;A_{G}=\frac{G_{V}-G_{R}}{G_{V}+G_{R}},\;\;A^{U}=\frac{B_{V}}{B_{R}}+5\frac{G_{V}}{G_{R}}-6. \end{array}$$

Moreover, the anisotropy of bulk modulus along the [100] and [001] directions, i.e., $B_{\left[100\right]}$ and $B_{\left[001\right]}$ are given by

(4) $$\begin{array}{c} B_{\left[100\right]}=\frac{1}{S_{11}+S_{12}+S_{13}},\;\;B_{\left[001\right]}=\frac{1}{2S_{13}+S_{33}}. \end{array}$$

The anisotropy of Young’s modulus along the [100], [001], [110] and [111] directions, i.e., $E_{\left[100\right]}$, $E_{\left[001\right]}$, $E_{\left[110\right]}$ and $E_{\left[111\right]}$ are given by

(5) $$\begin{array}{c} E_{\left[100\right]}=\frac{1}{S_{11}},\;\;E_{\left[001\right]}=\frac{1}{S_{33}},\;\;E_{\left[110\right]}=\frac{4}{2S_{11}+S_{12}+S_{66}},\\ E_{\left[111\right]}=\frac{9}{\left(2S_{11}+S_{33}\right)+2\left(S_{12}+2S_{13}\right)+\left(2S_{44}+S_{66}\right)}. \end{array}$$

In addition, the longitudinal ($v_{l}$) and transverse ($v_{t}$) sound velocities for the [100], [001] and [110] directions are given by [42]

(6) $$\begin{array}{c} \left[100\right]v_{l}=\left[010\right]v_{l}=\sqrt{\frac{C_{11}}{\rho }},\;\;\left[001\right]v_{l}=\sqrt{\frac{C_{33}}{\rho }},\;\;\left[001\right]v_{t1}=\sqrt{\frac{C_{44}}{\rho }},\\ v_{t1}=\left[010\right]v_{t2}=\sqrt{\frac{C_{66}}{\rho }},\;\;\left[110\right]v_{l}=\sqrt{\frac{C_{11}+C_{12}+2C_{66}}{2\rho }},\;\;\left[1\bar{1}0\right]v_{t2}=\sqrt{\frac{C_{11}-C_{12}}{2\rho }}, \end{array}$$

where $v_{t_{1}}$ and $v_{t_{2}}$ refer to the first and the second transverse mode of the sound velocity, respectively, and ρ is the mass density of the crystal. The minimum thermal conductivity ($k_{min}$) is further obtained by [43]

(7) $$\begin{array}{c} k_{min}=\frac{k_{B}}{2.48}n_{v}^{\frac{2}{3}}\left(v_{l}+v_{t_{1}}+v_{t_{2}}\right), \end{array}$$

where $n_{v}$ represents the number of density of atoms per volume. Because the total thermal conductivity is already treated as the summation of one longitudinal and two transverse acoustic branches, the equation might be suitable to study the anisotropic thermal conductivities of the crystal. The polycrystal longitudinal ($V_{L}$) and transverse ($V_{T}$) elastic wave velocities are given by [44]

(8) $$\begin{array}{c} V_{L}=\sqrt{\frac{3B+4G}{3\rho }},\;\;V_{T}=\sqrt{\frac{G}{\rho }}. \end{array}$$

The average elastic wave velocity ($V_{M}$) is given by [45]

(9) $$\begin{array}{c} V_{M}={\left[\frac{1}{3}\left(\frac{1}{V_{L}^{3}}+\frac{2}{V_{T}^{3}}\right)\right]}^{-\frac{1}{3}}. \end{array}$$

The Debye temperature ($\Theta_{D}$) is given by [45]

(10) $$\begin{array}{c} \Theta_{D}=\frac{h}{k_{B}}{\left[\frac{3n}{4\pi }\left(\frac{N_{A}\rho }{M}\right)\right]}^{\frac{1}{3}}V_{M}, \end{array}$$

where *h* is the Plank constant, $k_{B}$ is the Boltzmann constant, *n* is the number of atoms in the molecule formula, $N_{A}$ is the Avogadro’s number, and *M* the molecular weight.

## 3. Results and Discussion

### 3.1. Bulk Properties at Zero Pressure

The calculated lattice and elastic constants of Ti${}_{4}$Nb${}_{3}$Al${}_{9}$ phase at 0 GPa are presented in Table 1. Experimentally, the lattice constants *a* and *c* of Ti${}_{4}$Nb${}_{3}$Al${}_{9}$ phase were determined in the range of 5.58–5.84 Å and 8.15–8.45 Å from the selected area electron diffraction pattern [4,5], and were measured to be 5.607 Å and 8.270 Å from the X-ray diffraction pattern [3]. Theoretically, the lattice constants were calculated as a=5.651 Å and c=8.205 Å by the first-principles method, and the elastic constants were estimated from the strain–energy relationship as $C_{11}=222.71$ GPa, $C_{12}=60.27$ GPa, $C_{13}=87.99$ GPa, $C_{33}=187.36$ GPa, $C_{44}=104.77$ GPa and $C_{66}=23.06$ GPa [17]. These previous results are also presented in Table 1 for comparison. It is obvious that our results are in good agreement with previous experimental and theoretical data, showing that the present methods are reliable. For the specific case of tetragonal crystals, the conditions of mechanical stability at zero pressure are as follows [46]:

(11) $$\begin{array}{c} C_{11}-\left|C_{12}\right|>0,\;\;C_{33}\left(C_{11}+C_{12}\right)-2C_{13}^{2}>0,\;\;C_{44}>0,C_{66}>0. \end{array}$$

Obviously, the obtained elastic constants of Ti${}_{4}$Nb${}_{3}$Al${}_{9}$ phase can obey the above mechanical stability conditions, showing the tetragonal phase are mechanically stable at zero pressure. Actually, $\gamma_{1}$-Ti${}_{4}$Nb${}_{3}$Al${}_{9}$ has been experimentally observed as a precipitated stable phase in high Nb containing γ-TiAl-based alloys [3,7].

### 3.2. Pressure Dependence of Structural Property

**Figure 2:**
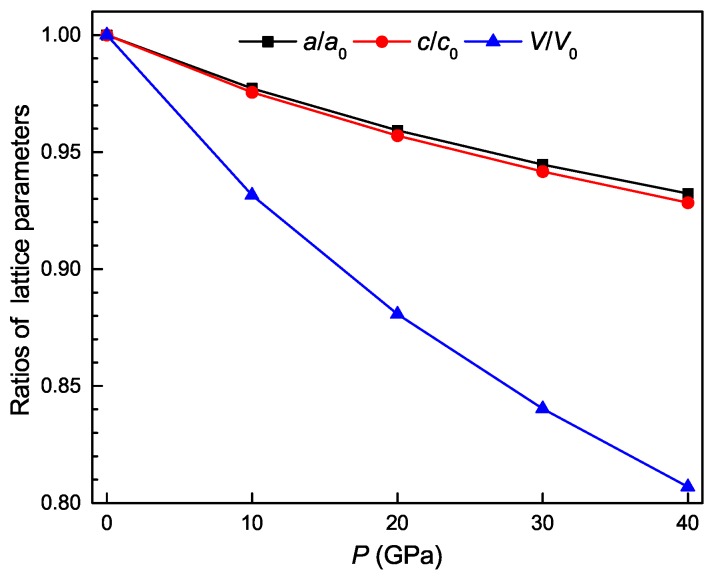
The ratios of lattice parameters as a function of pressure for the Ti${}_{4}$Nb${}_{3}$Al${}_{9}$ phase.

The optimized structural parameters of Ti${}_{4}$Nb${}_{3}$Al${}_{9}$ phase under different pressures are presented in Table 2. From the table, it is clearly seen that the lattice constants decrease with increasing the pressure, causing that the volume of unit cell shrinks and thus the mass density increases with increasing the pressure. The *c*/*a* values slightly decrease with increasing the pressure, indicating the better resistance against compression along the *a*-axis. The calculated normal lattice parameters *a*/$a_{0}$, *c*/$c_{0}$ and the normal volume *V*/$V_{0}$ of Ti${}_{4}$Nb${}_{3}$Al${}_{9}$ phase under different pressures are shown in Figure 2, where $a_{0}$, $c_{0}$ and $V_{0}$ correspond to the equilibrium lattice constants and volume at zero pressure. From the figure, it is clearly observed that all three normal parameters decrease with increasing the pressure. Meanwhile, the normal lattice parameter *a*/$a_{0}$ decreases more slowly than the counterpart *c*/$c_{0}$, showing the better resistance against compression along the *a*-axis.

### 3.3. Pressure Dependence of Elastic Properties

**Figure 3:**
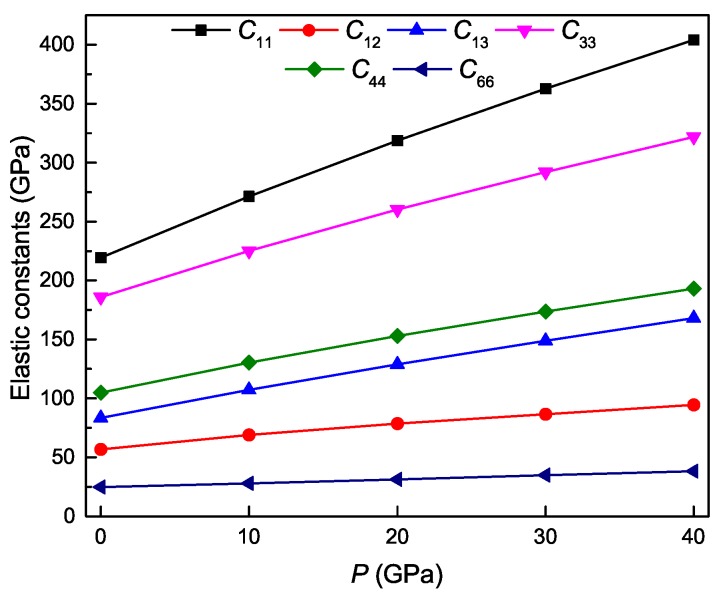
Elastic constants as a function of pressure for the Ti${}_{4}$Nb${}_{3}$Al${}_{9}$ phase.

The calculated elastic constant of Ti${}_{4}$Nb${}_{3}$Al${}_{9}$ phase under different pressures are shown in Figure 3. It is found that the six independent elastic constants of the phase increase monotonously with increasing the pressure. Through fitting these theoretical data at 0 K and under different pressures to the quadratic polynomial, the obtained relations are as follows:

(12) $$\begin{array}{c} C_{11}=219.54939+5.31161\times P-1.761\times 10^{-2}\times P^{2},\\ C_{12}=57.10825+1.21952\times P-7.26\times 10^{-3}\times P^{2},\\ C_{13}=83.58492+2.42181\times P-7.79\times 10^{-3}\times P^{2},\\ C_{33}=186.16486+4.01049\times P-1.558\times 10^{-2}\times P^{2},\\ C_{44}=105.02977+2.61051\times P-1.0321\times 10^{-2}\times P^{2},\\ C_{66}=24.67078+3.309\times 10^{-1}\times P-1.47371\times 10^{-4}\times P^{2}. \end{array}$$

It is evident that as the pressure increases, the constant $C_{11}$ varies most rapidly, followed by $C_{33}$, $C_{44}$, $C_{13}$ and $C_{12}$, and $C_{66}$ varies most slowly. It is also found that the theoretical value of $C_{11}$ is larger than that of $C_{33}$ under identical pressure, indicating that the strength of the bonding along the [100] and [010] directions is stronger than that along the [001] direction, and thus it is more difficult to compress along the [100] and [010] directions than along the [001] direction. Similarly, the theoretical value of $C_{44}$ is also larger than that of $C_{66}$ at identical pressure, indicating that the shear deformation along the [100](010) direction is easier than that along the [100](001) direction.

It is known that the mechanical stability conditions of the tetragonal crystal under isotropic pressure are as follows [46,47]:

(13) $$\begin{array}{c} {\tilde{C}}_{11}-\left|{\tilde{C}}_{12}\right|>0,\;\;{\tilde{C}}_{33}\left({\tilde{C}}_{11}+{\tilde{C}}_{12}\right)-2{\tilde{C}}_{13}^{2}>0,\;\;{\tilde{C}}_{44}>0,\;\;{\tilde{C}}_{66}>0, \end{array}$$

with

(14) $$\begin{array}{c} {\tilde{C}}_{ii}=C_{ii}-P\;\left(i=1,3,4,6\right),\;\;{\tilde{C}}_{12}=C_{12}+P,\;\;{\tilde{C}}_{13}=C_{13}+P. \end{array}$$

**Figure 4:**
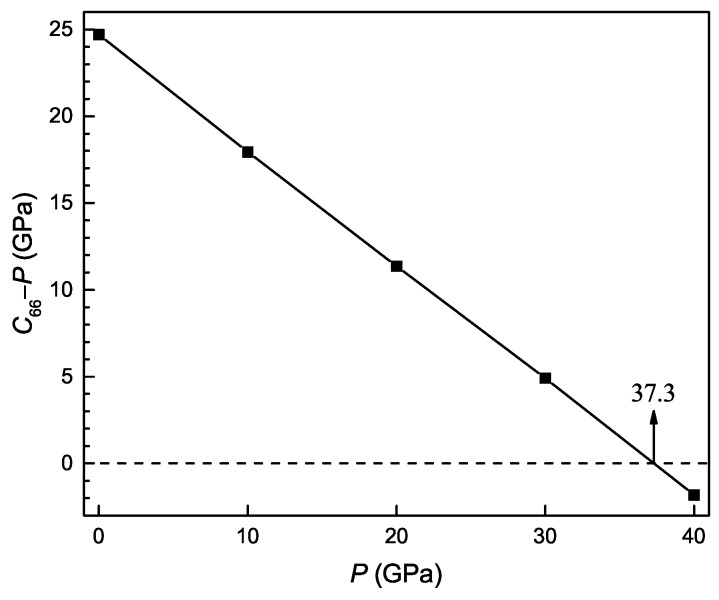
Difference between elastic constant $C_{66}$ and pressure *P* as a function of pressure for the Ti${}_{4}$Nb${}_{3}$Al${}_{9}$ phase.

The pressure dependence of the constant ${\tilde{C}}_{66}$ is shown in Figure 4 for the Ti${}_{4}$Nb${}_{3}$Al${}_{9}$ phase. It is seen that the value of ${\tilde{C}}_{66}$ is less than 0 when the pressure is beyond about 37.3 GPa, indicating that the tetragonal structure of the phase becomes mechanically unstable above pressures about 37.3 GPa.

**Figure 5:**
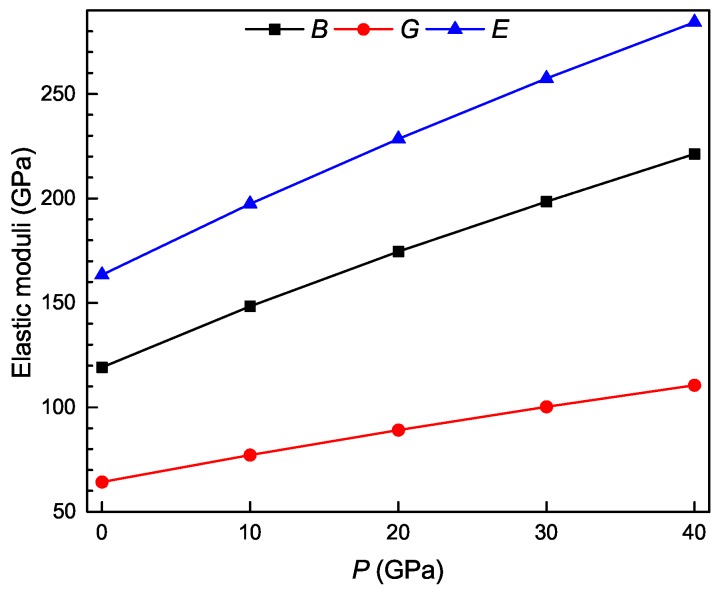
Bulk (*B*), shear (*G*) and Young’s (*E*) moduli as a function of pressure for the Ti${}_{4}$Nb${}_{3}$Al${}_{9}$ phase.

**Figure 6:**
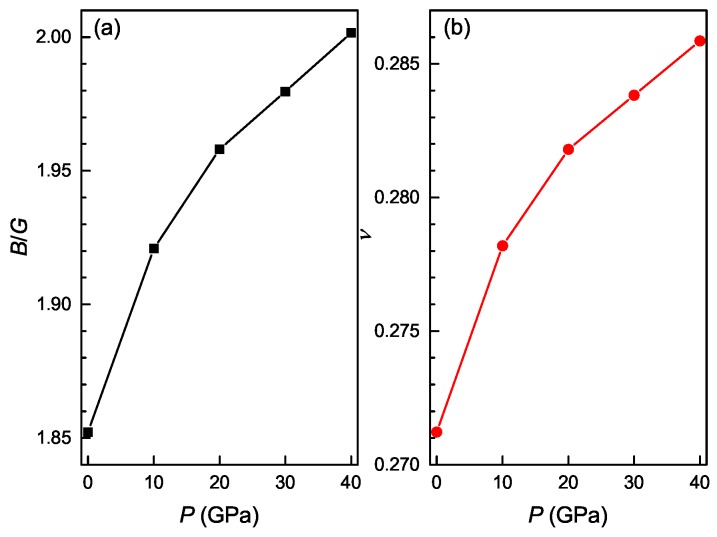
Ratio of bulk modulus to shear modulus (**a**) and Poisson’s ratio (**b**) as a function of pressure for the Ti${}_{4}$Nb${}_{3}$Al${}_{9}$ phase.

The bulk modulus *B* measures the resistance of a crystal to volume change. The shear modulus *G* measures the resistance of a crystal to shape change. The Young’s modulus *E* is defined as the ratio of tensile stress to tensile strain and often employed to provide a measure of the stiffness of a crystal. The larger the value of *E*, the stiffer the material. Figure 5 shows the calculated polycrystalline elastic moduli of the Ti${}_{4}$Nb${}_{3}$Al${}_{9}$ phase under different pressures. It is clear that the bulk modulus, shear modulus and Young’s modulus linearly increase with increasing the pressure, indicating that the phase has the enhanced resistance against volume change and shape change, and becomes stiffer as the pressure increases. Pugh [48] has introduced the ratio of bulk modulus to shear modulus (B/G) to assess the ductile/brittle behavior of a material. A high value of B/G corresponds to ductile nature, whereas a low value is correlated with brittleness. If B/G>1.75, the material behaves in a ductile manner; otherwise, it behaves in a brittle manner. The calculated values of B/G for the Ti${}_{4}$Nb${}_{3}$Al${}_{9}$ phase are shown as a function of pressure in Figure 6a. It is evident that the B/G ratio is always more than 1.75 and increases with increasing the pressure, indicating that the ductility of the phase is improved with the increase of pressure. Frantsevich et al. [49] has introduced the Poisson’s ratio to assess the ductile/brittle behavior of a material. If ν<0.26, the material exhibits a brittle manner; otherwise, it exhibits a ductile manner. The calculated Poisson’s ratios of Ti${}_{4}$Nb${}_{3}$Al${}_{9}$ phase are shown as a function of pressure in Figure 6b. It is evident that the Poisson’s ratio is always more than 0.26 and increases with increasing the pressure, also indicating that the ductility of the phase is improved as the pressure increases.

**Figure 7:**
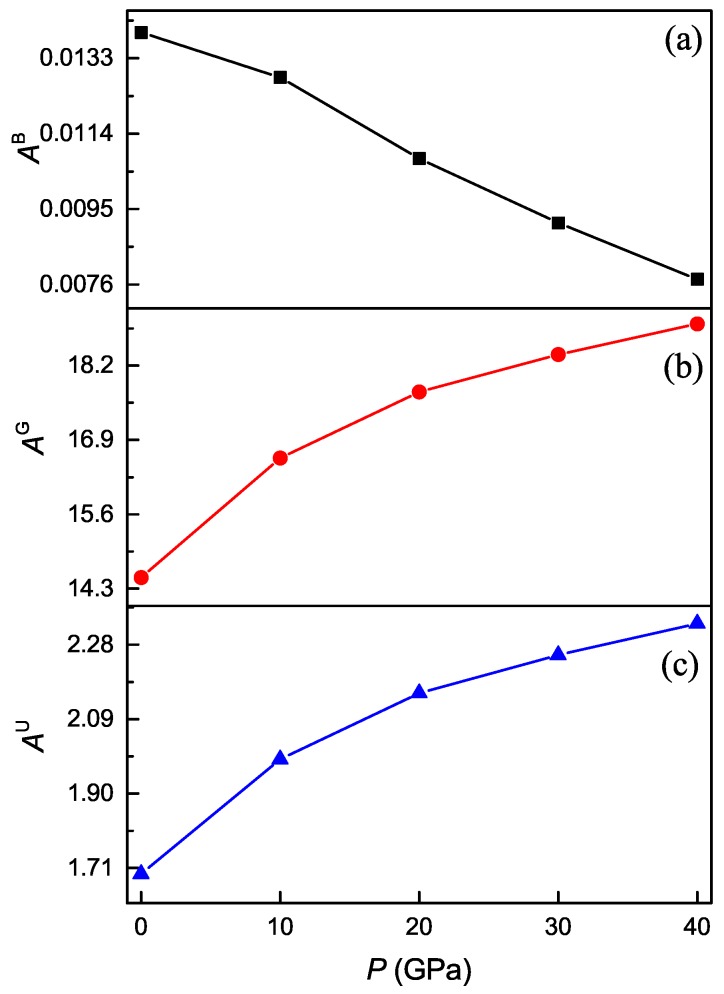
Percentage anisotropy in compressibility (**a**) and shear (**b**), and universal anisotropy (**c**) as a function of pressure for the Ti${}_{4}$Nb${}_{3}$Al${}_{9}$ phase.

**Figure 8:**
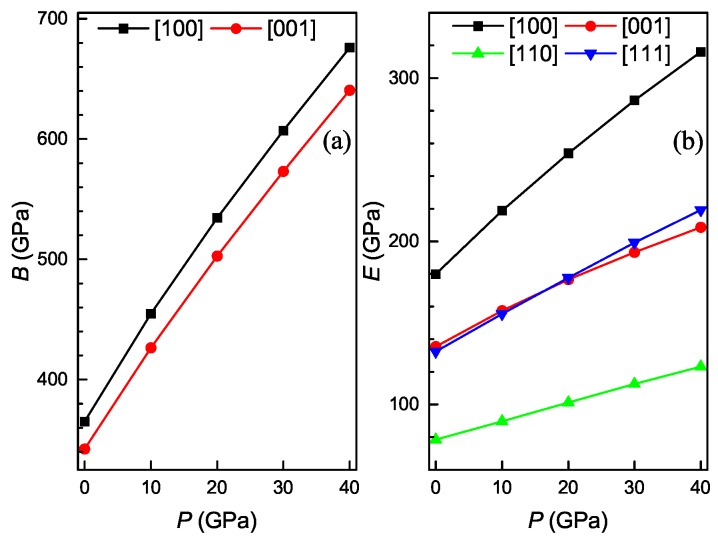
Directional bulk modulus (**a**) and Young’s modulus (**b**) along principle crystallographic axes as a function of pressure for the Ti${}_{4}$Nb${}_{3}$Al${}_{9}$ phase.

It is well known that any crystals are elastically anisotropic, and thus the measurement of elastic anisotropy is very important in the area of both crystal physics and engineering science. Figure 7 shows the calculated values of various anisotropy factors for the Ti${}_{4}$Nb${}_{3}$Al${}_{9}$ phase under different pressures. For an isotropic crystal, the percentage anisotropy factors $A^{B}$ and $A^{G}$ and the universal anisotropy $A^{U}$ must be equal to zero, while any departure from zero corresponds to the degree of elastic anisotropy. From Figure 7a,b, one can see that the positive value of $A^{B}$ decreases and that of $A^{G}$ increases with increasing the pressure, and the $A^{B}$ value is remarkably smaller than the $A^{G}$ one at identical pressure. These show that the anisotropy in compressibility weakens while the anisotropy in shear strengthens as the pressure increases for Ti${}_{4}$Nb${}_{3}$Al${}_{9}$ phase, and the shear anisotropy is more significant than the compressibility one. It is also seen from Figure 7c that the positive value of $A^{U}$ increases with increasing the pressure, showing that the anisotropy of the phase is enhanced with the increase of pressure. Moreover, the directional dependence of elastic moduli reflects the elastic anisotropy of a crystal. Figure 8 shows the calculated bulk and Young’s moduli along principle crystallographic axes for the Ti${}_{4}$Nb${}_{3}$Al${}_{9}$ phase under different pressures. From Figure 8a, one can find that the values of both $B_{\left[100\right]}$ and $B_{\left[001\right]}$ monotonously increase with increasing the pressure, and the $B_{\left[100\right]}$ value varies more rapidly than the $B_{\left[001\right]}$ one. At identical pressure, the value of of $B_{\left[100\right]}$ is always larger than that of $B_{\left[001\right]}$. It is found from Figure 8b that the values of $E_{\left[100\right]}$, $E_{\left[001\right]}$, $E_{\left[110\right]}$ and $E_{\left[111\right]}$ all increase with increasing the pressure, and the value of $E_{\left[100\right]}$ varies most rapidly, followed by $E_{\left[111\right]}$ and $E_{\left[001\right]}$, and $E_{\left[110\right]}$ varies most slowly. At identical pressure, the value of $E_{\left[100\right]}$ is always the largest while that of $E_{\left[110\right]}$ is always the smallest, and $E_{\left[111\right]}$ and $E_{\left[001\right]}$ have the same value at the pressure of about 16.5 GPa. These also show that the anisotropy of Ti${}_{4}$Nb${}_{3}$Al${}_{9}$ phase is enhanced with the increase of pressure.

### 3.4. Pressure Dependence of Acoustic and Related Properties

**Figure 9:**
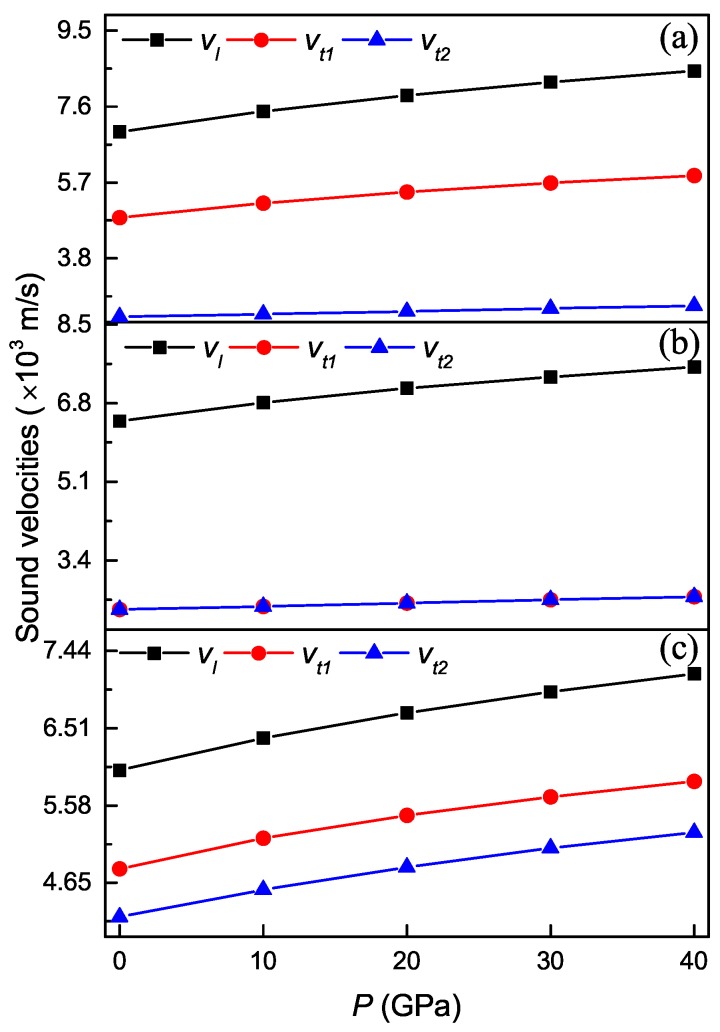
Longitudinal and transverse sound velocities in [100] (**a**), [001] (**b**) and [110] (**c**) directions as a function of pressure for the Ti${}_{4}$Nb${}_{3}$Al${}_{9}$ phase.

Acoustic velocities depend on the symmetry of crystal and the direction of propagation. For a specific case of tetragonal crystals, sound velocities only for the [100] (or [010]), [001] and [110] principle directions are pure longitudinal and transverse, while for all other directions they are either quasi-longitudinal or quasi-transverse. Figure 9 shows the calculated pure longitudinal and transverse sound velocities for various principle directions for the Ti${}_{4}$Nb${}_{3}$Al${}_{9}$ phase under different pressures. It is clear that both longitudinal and transverse sound velocities monotonously increase with increasing the pressure for all the three principle directions, and the longitudinal sound velocity for the [100] direction changes most rapidly, followed by [001], and that for the [110] direction changes most slowly. Meanwhile, the longitudinal sound velocity changes more rapidly than the corresponding transverse sound velocities. At identical pressure, the longitudinal sound velocity for the [100] direction is always the largest, followed by [001], and that for the [100] direction is always the smallest. These indicate that the anisotropy of acoustic velocities is enhanced with the increase of pressure for the Ti${}_{4}$Nb${}_{3}$Al${}_{9}$ phase.

**Figure 10:**
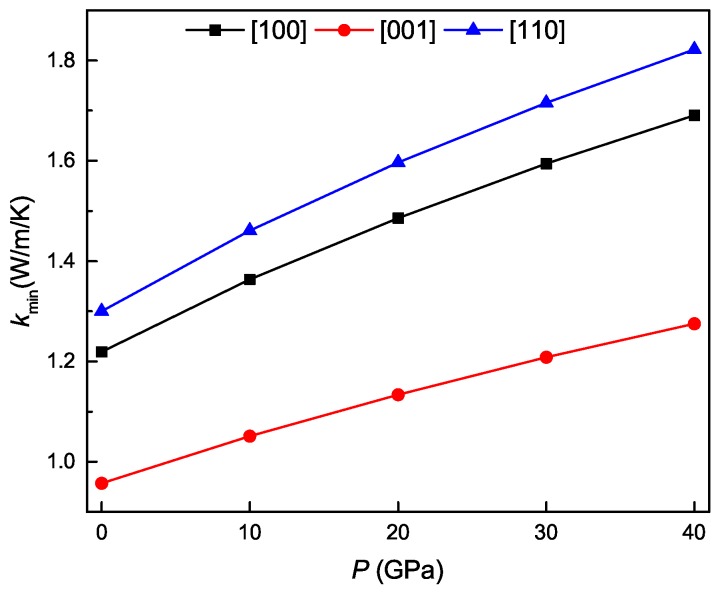
Minimum thermal conductivities in [100], [001] and [110] directions as a function of pressure for the Ti${}_{4}$Nb${}_{3}$Al${}_{9}$ phase.

The thermal conductivity of crystals is an important parameter. As temperature elevates, the thermal conductivity can reduce to a limiting value named minimum thermal conductivity. Figure 10 show the calculated minimum thermal conductivities in the [100], [001] and [110] directions for the Ti${}_{4}$Nb${}_{3}$Al${}_{9}$ phase under different pressures. It is obvious that the value of $k_{min}$ monotonously increases with increasing the pressure for all three of the directions, and the minimum thermal conductivity in the [110] direction varies most rapidly, followed by [100], and that in the [001] direction changes most slowly. At identical pressure, the largest minimum thermal conductivity is always in the [110] direction, followed by [001], and the smallest minimum thermal conductivity is always in the [100] direction. These indicate that the anisotropy of minimum thermal conductivity is enhanced with the increase of pressure for the Ti${}_{4}$Nb${}_{3}$Al${}_{9}$ phase.

**Figure 11:**
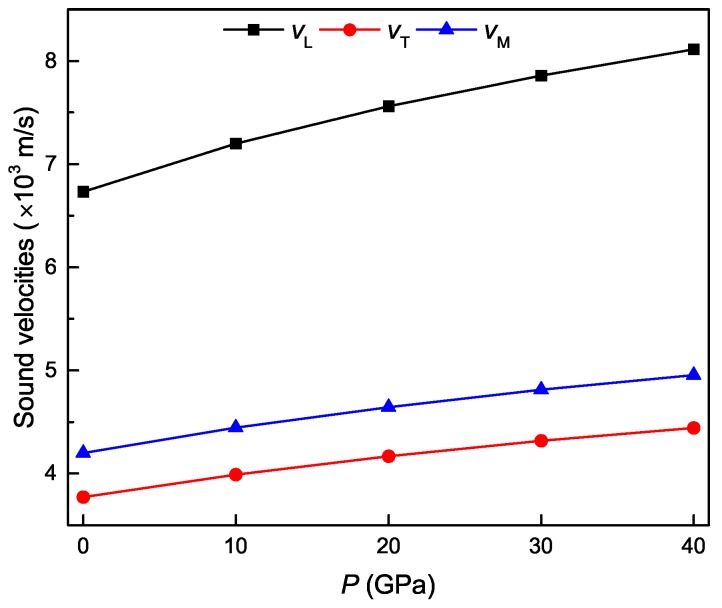
Polycrystal longitudinal, transverse and average sound velocities as a function of pressure for the Ti${}_{4}$Nb${}_{3}$Al${}_{9}$ phase.

**Figure 12:**
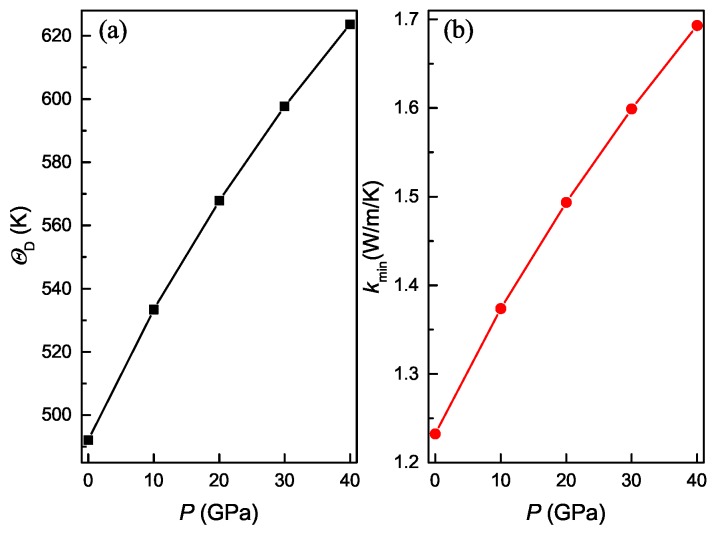
Polycrystal Debye temperature (**a**) and minimum thermal conductivity (**b**) as a function of pressure for the Ti${}_{4}$Nb${}_{3}$Al${}_{9}$ phase.

The calculated polycrystal longitudinal, transverse and average sound velocities for the Ti${}_{4}$Nb${}_{3}$Al${}_{9}$ phase under different pressures are shown in Figure 11. It is evident that both longitudinal and transverse sound velocities monotonously increase with increasing the pressure, and the longitudinal sound velocity varies more rapidly than the transverse sound velocity. At identical pressure, the longitudinal sound velocity is always larger than the corresponding transverse sound velocity. It is also observed that the average sound velocity monotonously increase with increasing the pressure, and its variation depends on the longitudinal and transverse sound velocities. Figure 12 show the calculated polycrystal Debye temperature and minimum thermal conductivity for the Ti${}_{4}$Nb${}_{3}$Al${}_{9}$ phase under different pressures. It is clear that the values of both $\Theta_{D}$ and $k_{min}$ monotonously increase with increasing the pressure, and exhibit a similar variation trend. These results obey the Callaway–Debye theory [50], in which the lattice thermal conductivity is proportional to Debye temperature.

## 4. Conclusions

The structural property and anisotropic elasticity of $\gamma_{1}$-Ti${}_{4}$Nb${}_{3}$Al${}_{9}$ phase under different pressures up to 40 GPa have been investigated by means of first-principles calculations. The obtained equilibrium structural parameters and elastic constants at 0 GPa are very consistent with the available experimental and theoretical values. The pressure dependent structural property and elastic constants have been presented. From the obtained high pressure elastic constants, the $\gamma_{1}$-Ti${}_{4}$Nb${}_{3}$Al${}_{9}$ phase is predicted to be unstable at the pressures above 37.3 GPa. The pressure dependence of elastic moduli, anisotropic factors, acoustic velocities, minimum thermal conductivities and Debye temperature have also been presented. As the pressure increases, the ductility of the $\gamma_{1}$-Ti${}_{4}$Nb${}_{3}$Al${}_{9}$ phase is found to be improved, and the anisotropy of elastic and related properties and Debye temperature are enhanced. The results shall be useful for future work.

## Figures and Tables

**Table 1:** Calculated lattice constants *a*, *c* (in Å) and elastic constants (in GPa) of Ti${}_{4}$Nb${}_{3}$Al${}_{9}$ at zero pressure.

Method	*a*	*c*	$C_{11}$	$C_{12}$	$C_{13}$	$C_{33}$	$C_{44}$	$C_{66}$
Present	5.6510	8.2051	219.36	56.83	83.43	185.91	104.86	24.71
Exp. [4,5]	5.58–5.84	8.15–8.45						
Exp. [3]	5.607	8.270						
Theo. [17]	5.651	8.205	222.71	60.27	87.99	187.36	104.77	23.06

**Table 2:** Optimized lattice parameters *a*, *c* (in Å), *c*/*a*, cell volume (in Å${}^{3}$) and mass density ρ (in g/cm${}^{3}$) of Ti${}_{4}$Nb${}_{3}$Al${}_{9}$ under different pressures.

*P*	*a*	*c*	c/a	*V*	ρ
0	5.6510	8.2051	1.4520	262.02	4.5194
10	5.5224	8.0042	1.4494	244.10	4.8511
20	5.4209	7.8525	1.4486	230.75	5.1317
30	5.3379	7.7269	1.4475	220.16	5.3785
40	5.2685	7.6168	1.4457	211.42	5.6009
